# Emergency Department Access During COVID-19: Disparities in Utilization by Race/Ethnicity, Insurance, and Income

**DOI:** 10.5811/westjem.2021.1.49279

**Published:** 2021-04-28

**Authors:** Jason Lowe, Ian Brown, Ram Duriseti, Moises Gallegos, Ryan Ribeira, Elizabeth Pirrotta, N. Ewen Wang

**Affiliations:** Stanford University School of Medicine, Department of Emergency Medicine, Palo Alto, California

## Abstract

**Introduction:**

In March 2020, shelter-in-place orders were enacted to attenuate the spread of coronavirus 2019 (COVID-19). Emergency departments (EDs) experienced unexpected and dramatic decreases in patient volume, raising concerns about exacerbating health disparities.

**Methods:**

We queried our electronic health record to describe the overall change in visits to a two-ED healthcare system in Northern California from March–June 2020 compared to 2019. We compared weekly absolute numbers and proportional change in visits focusing on race/ethnicity, insurance, household income, and acuity. We calculated the z-score to identify whether there was a statistically significant difference in proportions between 2020 and 2019.

**Results:**

Overall ED volume declined 28% during the study period. The nadir of volume was 52% of 2019 levels and occurred five weeks after a shelter-in-place order was enacted. Patient demographics also shifted. By week 4 (April 5), the proportion of Hispanic patients decreased by 3.3 percentage points (pp) (P = 0.0053) compared to a 6.2 pp increase in White patients (P = 0.000005). The proportion of patients with commercial insurance increased by 11.6 pp, while Medicaid visits decreased by 9.5 pp (P < 0.00001) at the initiation of shelter-in-place orders. For patients from neighborhoods <300% federal poverty levels (FPL), visits were −3.8 pp (P = 0.000046) of baseline compared to +2.9 pp (P = 0.0044) for patients from ZIP codes at >400% FPL the week of the shelter-in-place order. Overall, 2020 evidenced a consistently elevated proportion of high-acuity Emergency Severity Index (ESI) level 1 patients compared to 2019. Increased acuity was also demonstrated by an increase in the admission rate, with a 10.8 pp increase from 2019. Although there was an increased proportion of high-acuity patients, the overall census was decreased.

**Conclusion:**

Our results demonstrate changing ED utilization patterns circa the shelter-in-place orders. Those from historically vulnerable populations such as Hispanics, those from lower socioeconomic areas, and Medicaid users presented at disproportionately lower rates and numbers than other groups. As the pandemic continues, hospitals should use operations data to monitor utilization patterns by demographic, in addition to clinical indicators. Messaging about availability of emergency care and other services should include vulnerable populations to avoid exacerbating healthcare disparities.

## BACKGROUND

As the first cases of coronavrus 2019 (COVID-19) spread in municipalities across the United States, hospitals and healthcare teams prepared to receive a predicted influx of infected and acutely ill patients. Concurrently, state and local governments disseminated shelter-in-place and personal hygiene recommendations hoping to mitigate rates of transmission and attenuate surges in patient volume. Northern California was one of the first areas in the US to identify community cases of COVID-19. 1 On March 16, 2020, the six Bay Area public health officers announced a shelter-in-place order. On March 19 California Governor Gavin Newsom announced a statewide “stay at home” order. These orders mandated that citizens should remain at home for all but “essential duties” and minimize interpersonal contact. News outlets and other media broadcast this information widely.

Our health system enacted measures in preparation for a potential increase in patients and heightened resource utilization to our two suburban emergency departments (ED). Elective surgeries and procedures were cancelled or postponed in an effort to reduce contact with infected individuals and decrease consumption of resources such as personal protective equipment (PPE). Telemedicine systems were encouraged and enhanced systemwide. Messaging and workflows were also developed to direct non-emergent visits to other care sites. These measures, coupled with the shelter-in-place order, led to a rapid change in ED census and a notable decline in overall visits.

While a few localities were overwhelmed by high numbers of severely ill patients, many EDs in the nation experienced a sudden drop-off in patient volumes.^2^–^4^ As the COVID-19 pandemic evolved, public health concerns shifted to include worries that individuals with life-threatening conditions were avoiding the ED, leading to delayed presentations and negative outcomes. 2,3 Others have found that fear of contracting COVID-19 and obeying the shelter-in-place orders were significant reasons that patients avoided the ED, but a detailed demographic breakdown was not performed. 4 Further weeks into the pandemic, minorities, particularly Black and Hispanic, were noted to have disproportionately higher incidences of hospitalizations and deaths due to COVID-19 vs other groups. 5,6

Historically, researchers have found that external forces such as natural disasters, weather patterns, holidays, and other major events can affect access to care and healthcare utilization patterns. 7–11 However, no acute societal event in recent times has had the scope or duration of the COVID-19 pandemic. Thus, there is little understanding of how perceptions of ED access changed or of the resulting utilization by different patient demographic populations. While the initial shelter-in-place order had a deadline of March 28, it was extended with modifications using a phased approach. 12 This extension has contributed to prolonged alterations in ED patient volume and characteristics. Even once official orders end, attempts to decrease social contact will likely continue; the move to telemedicine may be enduring and there will be subsequent fluctuations in COVID-19 cases. Thus, the response to the pandemic will likely have continuing and unpredictable effects on ED and hospital volume, access, and utilization.

**Population Health Research Capsule**

What do we already know about this issue?

*Directives to minimize transmission of coronavirus disease 2019 (COVID-19) caused drastic alterations in emergency department (ED) visits.*

What was the research question?

*We sought to characterize the impact of shelter-in-place orders on various demographic groups in our two EDs.*

What was the major finding of the study?

*Early on, high-risk groups presented to our EDs at a lower rate. Later, they presented in higher numbers, with higher acuity.*

How does this improve population health?

*Future deployment of services and messaging should be aimed at addressing the gaps found in access to healthcare services for high-risk populations.*

Our objective was to understand ED volume and utilization by patient socioeconomic characteristics during these dynamic times. We hypothesized that vulnerable populations would have decreased and altered utilization of the ED compared to the prior year.

## METHODS

We analyzed the electronic health record (EHR) data from the two EDs within our health system.

### Study Sites and Population

Our hospital system is located in the San Francisco Bay Area and is a national and statewide tertiary referral hospital. The combined county populations (Alameda, Santa Clara, San Mateo) are approximately 4.2 million, with an average household income ranging from $90,000–$115,000. The population is approximately 30–45% White (not Hispanic), 22–25% Hispanic, 30–40% Asian, and 2–11% Black. 13 Our hospital system consists of two hospitals and three ambulatory care settings. The first hospital is a large, suburban, quaternary referral center with approximately 80,000 ED visits a year located in Palo Alto, California. The second hospital is a suburban community hospital with approximately 37,000 ED visits a year, located in Pleasanton, California. In 2019 our combined hospitals’ ED population was 22% 0–17 years; 33% 18–44; and 22% 45–65 and >65 years; 39% White, 29% Hispanic, 16% Asian, and 7% Black (Table 1).

We analyzed all ED visits starting March 1, 2020, when changes to census numbers were first noted (two weeks before imposition of the shelter-in-place orders) and continued through June 30, 2020. We compared this time period to the identical period in 2019. A five-year review of hospital census data revealed no significant changes in demographics, affirming that 2019 represented an appropriate sample for comparison. Weeks were counted as the seven days from Sunday to Saturday. The week of March 15 was identified as Week 1, which was when our local shelter-in-place order was enacted.

### Key Timepoints Identified

Healthcare system stops elective procedures: March 13, 2020

Bay Area shelter-in-place order: March 16, 2020

California stay-at-home order: March 19, 2020

Healthcare system resumes elective procedures: May 4, 2020

### Demographic Characteristics

We grouped age into standard categories of < 18, 18–44, 45–64, and > 65 years. Gender was categorized as male and female. Insurance was grouped as commercial (private and worker’s compensation), Medicaid, Medicare, and self-pay. Reported race and ethnicity was grouped as Hispanic, Non-Hispanic-White, Black, Asian and Pacific Islander, and Other (including missing <1% and no answer). We were unable to use language as a variable due to lack of availability of reporting.

Census tract was determined from patient street address and then matched to median household income from the US Census American Community Survey 2018 five-year estimates. 14 We grouped median household income by comparison to 2018 federal poverty level (FPL) for a family of four ($25,100). 15 We used standard categories of <300% FPL, 300–400% FPL, and >400% FPL. For patients missing census tract information, ZIP codes were matched to median household income from 2006–2010 found at Tract2Zip (https://www.psc.isr.umich.edu/dis/census/Features/tract2zip/). Groupings were made using the 2010 FPLs for a family of four ($22,050). Thus, we were unable to assign an income level to only 3.5% of addresses.

### Clinical Characteristics

Acuity was represented by Emergency Severity Index (ESI) triage level. High-acuity trauma and ESI levels of 1 and 2 were categorized as “Resuscitation/Emergent”; low-acuity trauma and ESI level 3 were categorized as “Urgent.” We characterized ESI triage levels 4 and 5 “Semi-/Non-Urgent.” ED disposition was categorized as admission, discharge, transfer, expired, and against medical advice (AMA). Transferred, expired, and AMA categories occurred at least a level of magnitude less than admission and discharge and are not shown separately in our figures.

Primary *International Classification of Diseases, Revision 10* (ICD-10) diagnosis code was grouped using Healthcare Cost and Utilization Project (HCUP) Clinical Classification Software (CCS), 16 which resulted in 17 groups. These groupings were chosen to represent higher acuity diagnoses, which, if analyzed separately, would not have been large enough to show statistical significance. We identified the top five most populous code groups (circulatory, infection, injury, neurological, and respiratory), with the remaining groups aggregated as “Other.” Because each patient had only one primary diagnosis, the CCS categories are mutually exclusive.

### Calculations

We aggregated data by week for both study years. Frequency and proportions of each study variable were calculated by week. We also calculated by week the difference in proportions of patients with a given characteristic between 2020 and 2019. Frequency and percentage point (pp) change in proportions were compared on a timeline to evaluate trends. The z-score was calculated for a difference in proportions between 2019 and 2020 to identify whether the difference in rates was due to chance alone. We set the a priori significance level at *P* = .05. We used SAS statistical software v9.4 (SAS Institute, Inc., Cary, NC) for the data calculations; and we used interactive data visualization software (Tableau Software, LLC, Seattle, WA) for the data visualizations. Our institutional review board (IRB) determined this to be a quality improvement project and thus IRB exempt.

## RESULTS

**Figure 1:**
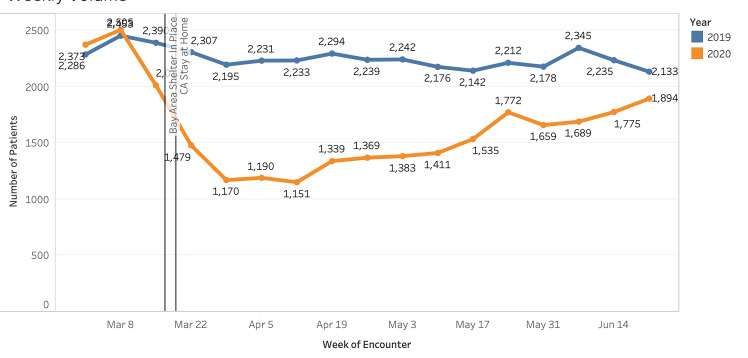
Overall weekly changes in emergency department volume in 2020 compared to 2019 (March 1–June 30 2020). *ED*, emergency department.

**Figure 2:**
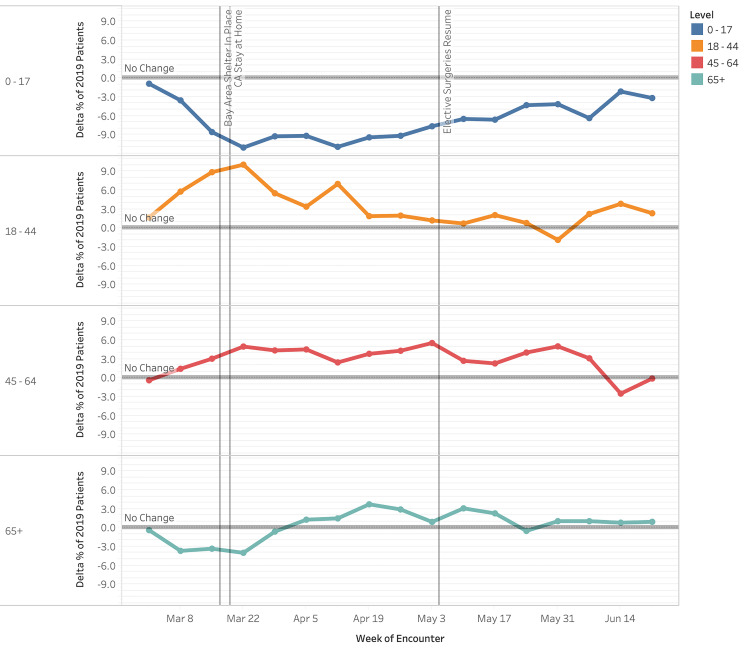
Change in proportion of patients by age (years) for weekly emergency department visits in 2020 compared to 2019 (March 1–June 30, 2020). *ED*, emergency department.

Emergency department volume decreased approximately 28% compared to the 2019 control period (27,706 visits vs 38,291 visits). A notable decrease in volume began the week prior to the Bay Area shelter-in-place order (March 8, 2020). This continued to its nadir (Week 5, April 12, 2020) and volume decreased 52% compared to 2019 (1,151 visits vs 2,233 visits) (Figure 1). Pediatric patients (0–17 years) experienced a proportionally large decline, with a 50% decrease during the study period vs the control period in 2019 (3,351 visits vs 6,700 visits [data not shown]). Visits among pediatric patients at Week 2 (March 22) were −11.0 pp compared to those from 2019 (Figure 1, *P = <* .00001). Visits among those 18–44, 45–64, and >65 years were +10.1 pp (*P* < .00001), +5.0 pp (*P <* .00001) and −4.0 pp (*P* = .00014) respectively at Week 2 (March 22) compared to those from 2019 (*P* < .05 for all changes). By Week 15 (June 21), weekly volumes for most age groups had returned to nearly 2019 levels, although children <17 years had a −3.2 pp change from baseline (*P =* 0.00023). The volumes by gender fluctuated weekly, with no consistent trend toward significance during the study period (*P values were not consistently <* .05). Figure 2 displays our findings of changes in proportion by age.

**Figure 3:**
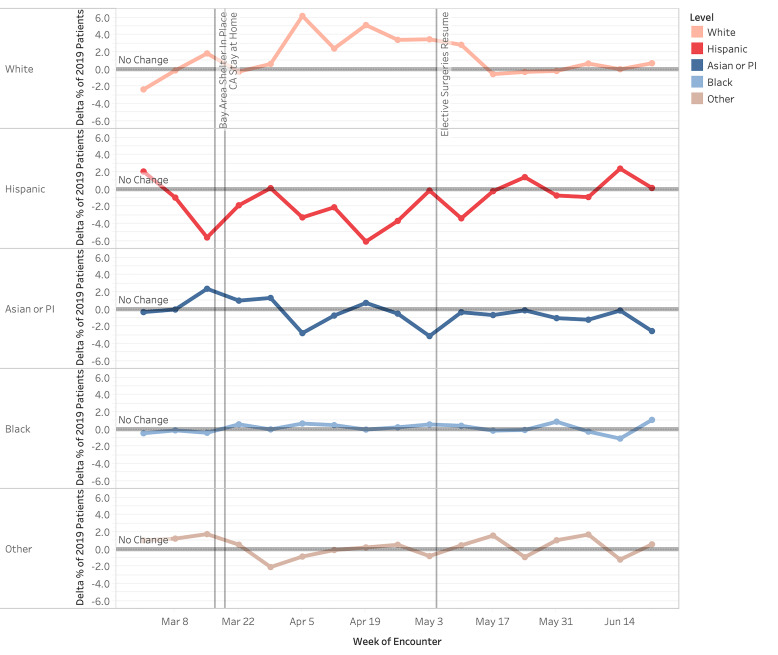
Change in proportion of patients by race/ethnicity for weekly emergency department visits in 2020 compared to 2019 (March 1–June 30, 2020). *ED*, emergency department.

Hispanic patient visits were proportionally decreased compared to 2019, while visits by White patients were increased (Figure 3). By Week 4 (April 5), Hispanic patient visits overall decreased 3.3 pp (*P* =.0054) compared to a 6.2 pp (P *<* .00001) increase by White patients. Hispanic visits experienced a nadir of −6.1 pp (*P <* .00001) in Week 6 (April 19). Asian and Pacific Islander and Black patients had fluctuating changes in proportion, which did not consistently trend to significance (*P values were not consistently <* .05).

**Figure 4:**
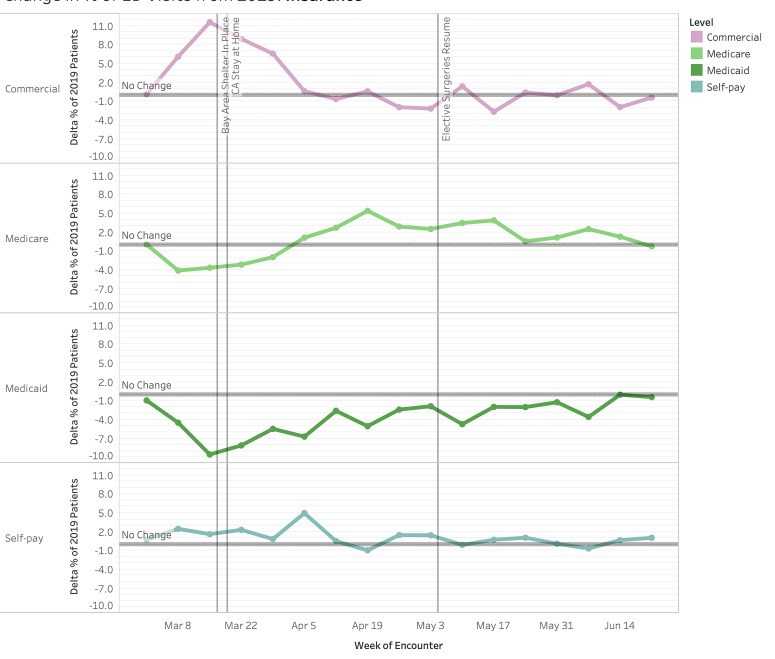
Change in proportion of patients by insurance status for weekly emergency department visits in 2020 compared to 2019 (March 1–June 30, 2020). *ED*, emergency department.

All payor categories trended toward statistically significant changes. The proportion of patients with commercial insurance started increasing the week before shelter-in-place, peaking at +11.6 pp at Week 1 of shelter-in-place (Figure 4, *P <* .00001), but returning to baseline proportions (*P* > .05) for most of the remaining weeks. The majority of this increase was offset by a decrease in Medicaid patients by −9.5 pp at Week 1 (*P <* .00001). This trended upward in subsequent weeks to reach equivalent proportions to 2019 at Week 14. Throughout the study period, the proportional decrease in Medicaid visits remained statistically significant. Medicare visits nadired the week before the shelter-in-place order at −4.1 pp (*P <* .00001) and gradually increased to +5.4 pp (*P <* .00001) change from 2019 visits in Week 4 (April 5). The fluctuations in Medicare visit proportion were statistically significant for the majority of weeks examined.

**Figure 5:**
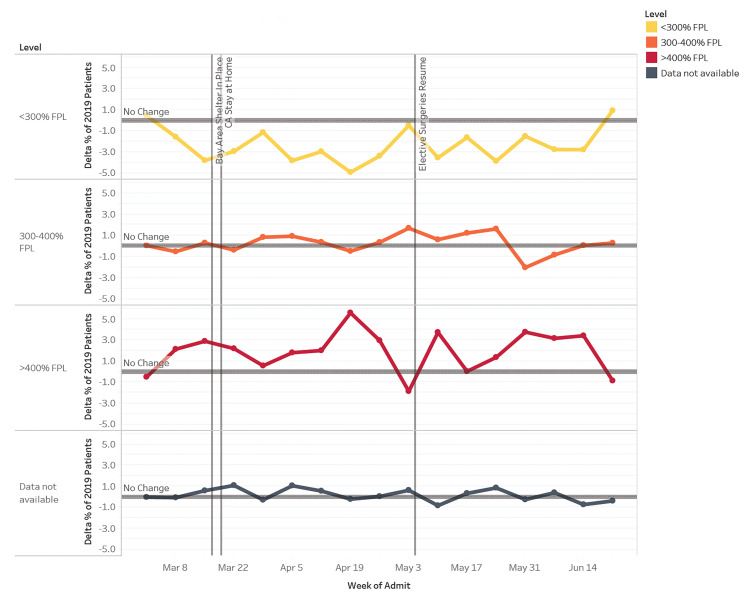
Change in proportion of patients by federal poverty level categories for weekly ED visits in 2020 compared to 2019. Federal poverty level = $25,100 (March 1–June 30, 2020). *ED*, emergency department.

The proportion of patient visits from addresses <300% FPL nadired at −3.8 pp (*P* = .000046) at week one of shelter in place (Figure 5); changes in proportion were statistically significant for 11 of the 15 weeks (*P* < .05) examined. In comparison, patient visits from census tracts at >400% FPL were at 2.9 pp (*P* =.0044) above baseline at Week 1 of shelter in place and generally stayed well above baseline for the remainder of the study period. However, these proportions were statistically significant for only eight of the weeks examined.

**Figure 6:**
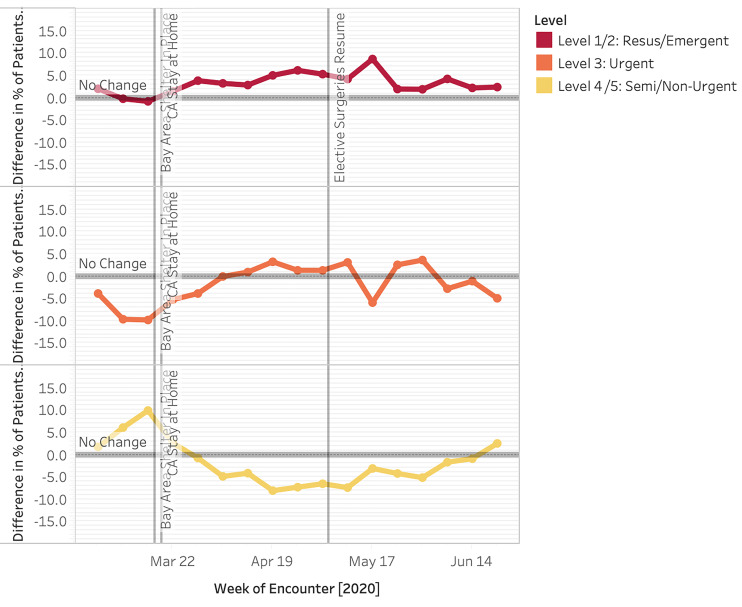

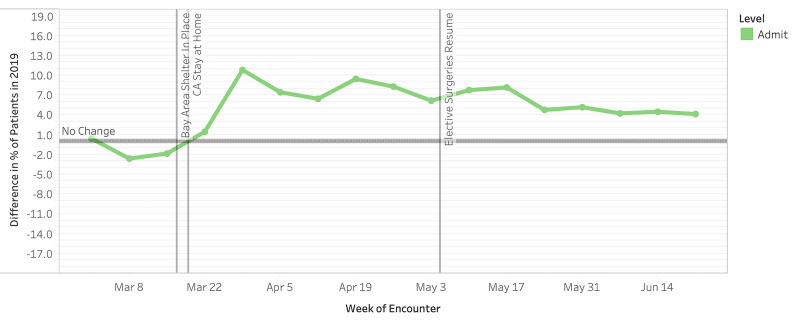
Change in proportion of patients by acuity as measured by Emergency Severity Index (ESI) level for weekly emergency department (ED) visits and percent change by admissions for weekly ED visits in 2020 (top) compared to 2019 (bottom) (March 1–June 30 2020). *ED*, emergency department.

We also analyzed visit acuity via the Emergency Severity Index (ESI) and admission rate (Figure 6). At the start of the study period, we observed an increase in lower acuity visits with a +10 pp peak in ESI level 4 and 5 patients at Week 1 of shelter in place (*P <* .00001). This was driven by a preponderance of COVID-19 testing requests as captured by “chief complaint” (data not shown – ICD-10 codes not existent). There was a consistently higher proportion of ESI level 1 patients throughout the study period vs the control period that was statistically significant for 13 of the 14 post shelter-in-place weeks examined (*P* = .0015 to *P <* .00001). During Week 10 (May 17), the proportion of ESI level 1 and 2 patients peaked at 8.7 pp vs 2019. However, due to the overall decrease in ED volume, the absolute number of ESI level 1 and 2 patients was 50% of 2019 levels at Week 5 after shelter in place and, overall, 79% 2019 levels.

Overall, the rate of admissions increased in 2020 (29.0% vs 24.7%) (Figure 6). Admission percent change peaked during Week 3, with a 10.7 pp increase compared to 2019; this attenuated at weeks 11–15 to approximately 4.5 pp. Admission percentage elevation above 2019 levels was statistically significant for all but one of the 14 post-shelter-in-place weeks examined (*P* = .000036 to *P <* .00001).

Additionally, we analyzed discharge diagnosis based on HCUP groupings. The circulatory diagnosis group proportion fluctuated throughout, with a slight trend toward an increase that reached statistical significance for 10 of the 15 weeks examined. Respiratory complaints rose sharply through Week 5 (April 12) (*P =* .0058 *to P <* .00001) and then declined. The drop in neurologic complaints reached statistical significance for all but two of the 15 weeks examined (*P* < .05), with a maximal drop of −4.6 pp (*P <* .00001) in Week 1 (March 15). Absolute numbers were decreased overall for all conditions when compared to 2019 levels (data available upon request).

## DISCUSSION

Our results uniquely demonstrate unreported disparities in ED utilization by historically vulnerable demographic populations due to COVID-19 and the shelter-in-place order. We found significantly reduced ED utilization patterns by race, ethnicity, payor-status, and household poverty groups during the study period. Similar to others, we also demonstrate changes in the absolute number of ED patients, as well as in the percent change in volume during the different phases of the pandemic response.^2^ Visit volumes fluctuated, first decreasing and then gradually increasing to almost baseline levels at the end of our study period. Percent change in patient acuity, measured by ESI and admissions, increased from 2020 compared to 2019; however, the absolute number of visits was still decreased.

Telemedicine services rose to prominence and may have played a role in the changes we and others have observed. In response to social distancing and shelter-in-place orders, our health delivery system underwent a drastic shift to telehealth visits for primary and specialty care services. The rapid development of protocols and infrastructure created new opportunity for patients to seek care via telehealth services. Historically, however, intervention-generated inequalities that further exacerbate disparities have been shown to arise from technologically related advances.^33^ Furthermore, a study of healthcare utilization in New York City at the early peak of the pandemic demonstrated that Black and Hispanic patients continued to use the ED and in-person office visits rather than telehealth.^34^ Similarly, many patients who seek emergency care in our healthcare system do not have access to outpatient care services due to insurance networks. The absolute decrease in ED utilization raises concern that while some patients were able to turn to telehealth or other avenues for alternate care, others may have been unable to. These questions beg additional study.

Our patient population consists of a large proportion of Hispanic patients. It has been reported that minority groups, specifically Hispanics and Blacks, have disproportionately higher morbidity and mortality rates due to COVID-19. 6 These groups historically experience decreased access to healthcare overall, even prior to the impact of COVID-19. 21–23 We demonstrate that initially Hispanics did not present to the ED for care at the same rate as White populations. We conjecture that this could have been due to a range of factors, including language barriers, lack of insurance, and misinformation about disease course. Additionally, some have noted that anti-immigrant policies and heightened immigration enforcement practice have caused increased immigrant fear of seeking healthcare. 24 This delayed presentation may be an additional factor influencing poorer outcomes, including deaths at home due to COVID-19 or for other medical reasons. 18–20

It is important to note that while our catchment area has a significant proportion of Asians (approximately 35%), 13 the Asian population using our EDs was only 16%. Additionally, our analyses show no statistically significant change in proportion of visits compared to 2019. Reasons for this lack of change may be due to cultural differences for seeking healthcare and/or the heterogeneous composition of the Asian ethnic grouping. We show minimal change in utilization for Black populations; however, our population size for this group was not sufficient to demonstrate disparities documented elsewhere.

Insurance type also influenced visit rates, with Medicaid patients initially presenting at a lower frequency than those who were commercially insured. While it is difficult to obtain data given that ICD-10 coding for COVID-19 was not uniform in the early stages of the pandemic, we postulate that use of the ED by patients with commercial insurance early in the pandemic could have been for COVID-19 testing as it corresponded to an increase in visits of lower severity and chief complaint. Later during the pandemic, decreased ED use by this same population may have been due to the fact that medical practices that cater to commercial insurance holders were able to adapt more rapidly and deploy solutions, such as telehealth visits, which deflected their patients from the ED. Conversely, we show that those patients using Medicaid presented to the ED less in the early weeks of the pandemic, but later presented more than patients with commercial insurance. This pattern of use raises concern for the underlying drivers to delays in seeking care. Patients who use these public programs have been ranked among the most vulnerable members of the US population, 25 and in California 60% of Medicaid enrollees are Hispanic 26,27 highlighting the multiple risk categories many patients straddle. With this concern in mind, efforts to target this population with accurate information and services, in the appropriate language, within their communities should be considered.

Household income level by census tract level also impacted ED visits. It is important to note that our catchment area includes wide disparities in economic status and that the cost-of-living renders FPL incomes untenable to survival here ($25,100 in 2018). Those from ZIP codes with incomes at >400% FPL had increased utilization of the ED, while those in ZIP codes with incomes <300% FPL had decreased utilization. Concerns over the cost of care, occupational demands, childcare needs, and lack of transportation are only some of the challenges that may have interdicted ED presentation at lower income levels. While insurance type, ethnicity/race, and economic status are likely intertwined, we were unable to make more than an observational relationship in our analyses.

Similar to multiple sites nationwide, we experienced a decline in absolute number of ED visits, 2,17 evidencing the intended effect of the shelter-in-place order. However, this initial decrease unexpectedly included more acute diagnoses such as myocardial infarction and stroke. 2 We also noted a decrease in absolute numbers of patients with high-acuity triage categories (ESI 1) and admissions. The prevalence of these emergent conditions should not be affected by COVID-19 or shelter-in-place orders and delays in their presentation could lead to higher morbidity and mortality as the conditions advance at home. This phenomenon may have occurred because warnings regarding COVID-19 exposure in the ED could have frightened some populations into not heeding serious signs and symptoms. 4,18–20 Due to sample size, we were unable to delve more deeply into the effects of insurance status or race/ethnicity on ESI levels. Further analysis is necessary to understand whether excess deaths that have occurred during the pandemic are due to COVID-19 vs other causes and also to understand which populations experienced these deaths.

While this is a study of one healthcare system with two distinct EDs, we suggest that generalized and standard EHR data assessment should include socioeconomic demographics and should be performed in a timely and regular fashion to ensure equitable utilization of services. Regional hospitals should pool data in order to give statistical power to understand and answer questions we were unable to answer, such as disparities in ED presentations of specific emergent conditions. For example, psychiatric diagnoses, domestic violence, and non-accidental trauma have been postulated to be at risk of increasing within certain populations during this current pandemic. 29–32

## LIMITATIONS

These results are limited to one hospital system with two distinct hospital EDs, which may reduce generalizability to other EDs. However, for expediency we used our institutional EHR to obtain real-time data regarding utilization during the pandemic; more representative, curated regional state or national data is more difficult and less timely to obtain, needing the cooperation of segregated systems. Additionally, the California Bay Area is unique in its racial/ethnic population mix 2,17 and differs from other states. The cost of living in our catchment area is higher than indicated by the FPL standards. Despite this, many of our clinical data analyses are qualitatively similar to those reported elsewhere.^2^,^3^,^6^,^17^

## CONCLUSION

Our study demonstrates the disparate impact that a global pandemic and shelter-in-place order has on ED utilization by various demographic groups. As the pandemic continues, disease surges as well as policy changes will further alter these patterns. Using electronic health record data, we can rapidly evaluate the systems in which we operate. Where before a chart review of demographic information would take analysts months to perform, we now have near real-time access. Healthcare systems can cross-reference admissions and operations data with demographic data to appreciate whether emergency care is being accessed and used equitably. With this information, we can target vulnerable populations using appropriate language and cultural awareness to reduce barriers to care in the ED and other medical resources. 2,3,28 This should include assurances that access to newer care modalities, such as telemedicine, are made available for all patient populations. Moving forward, this will be crucial to prevent widening disparities during the COVID-19 pandemic and general health outcomes in the future.

## Figures and Tables

**Table 1:** 2019 baseline demographics.

	N	%
Gender		
Female	20,045	52%
Male	18,503	48%
Age group		
0 – 17	8,481	22%
18 – 44	12,459	32%
45 – 64	8,538	22%
65+	8,813	23%
Race/ethnicity		
Black	2,533	7%
Other	4,265	11%
Asian or PI	6,307	16%
Hispanic	10,253	27%
White	14,933	39%
Insurance mix		
Commercial	15,180	40%
Medicare	9,139	24%
Medicaid	12,256	32%
Household income		
<300% FPL	8,044	21%
300–400% FPL	5,229	14%
>400% FPL	18,510	48%
Data not available	6,508	17%
ESI acuity level		
Levels 1 & 2: resus/emergent	6,390	17%
Level 3: urgent	22,703	59%
Levels 4 & 5: semi/non-urgent	8,660	23%
Not recorded	538	1%
Disposition		
Admitted	17,618	27%
Discharged	46,044	70%
Transfer	993	2%
AMA	1,249	2%

*PI*, Pacific Islander; *FPL*, federal poverty level; *AMA*, against medical advice.
